# Biocontrol Strategy of *Listeria monocytogenes* in Ready-to-Eat Pork Cooked Ham Using Peptic Hydrolysates of Porcine Hemoglobin

**DOI:** 10.3390/foods13152394

**Published:** 2024-07-29

**Authors:** Zain Sanchez-Reinoso, Sarah Todeschini, Jacinthe Thibodeau, Laila Ben Said, Ismail Fliss, Laurent Bazinet, Sergey Mikhaylin

**Affiliations:** 1 Institute of Nutrition and Functional Foods (INAF), Université Laval, Quebec City, QC G1V 0A6, Canada; zain.sanchez-reinoso.1@ulaval.ca (Z.S.-R.); sarah.todeschini.1@ulaval.ca (S.T.); jacinthe.thibodeau.1@ulaval.ca (J.T.); laila.ben-said.1@ulaval.ca (L.B.S.); ismail.fliss@fsaa.ulaval.ca (I.F.); laurent.bazinet@fsaa.ulaval.ca (L.B.); 2 Laboratory of Food Sustainability (EcoFoodLab), Food Science Department, Université Laval, Quebec City, QC G1V 0A6, Canada; 3 Laboratoire de Transformation Alimentaire et Procédés ÉlectroMembranaires (LTAPEM, Laboratory of Food Processing and Electromembrane Processes), Food Science Department, Université Laval, Quebec City, QC G1V 0A6, Canada; 4 International Associated Laboratory in Bioproduction of Natural Antimicrobials (LIAAN), Université Laval, Quebec City, QC G1V 0A6, Canada

**Keywords:** antimicrobial peptides, biopreservation, blood valorization, circular economy, hemoglobin

## Abstract

*Listeria monocytogenes* is a foodborne pathogen that represents a serious concern for ready-to-eat (RTE) meat products due to its persistence in production facilities. Among the different strategies for the control of this pathogen, the use of antimicrobial peptides derived from food by-products, such as slaughterhouse blood proteins, has emerged as a promising biocontrol strategy. This study evaluated for the first time the use of peptic hydrolysates of porcine hemoglobin as a biocontrol strategy of *L. monocytogenes* in RTE pork cooked ham. Pure porcine hemoglobin (Hb-P) and porcine cruor (P-Cru) were hydrolyzed using pepsin at different temperatures (37 °C for Hb-P and 23 °C for P-Cru) for 3 h. Then, the hydrolysates were characterized in terms of their degree of hydrolysis (DH), peptide population, color, and antimicrobial activity (in vitro and in situ) against three different serotypes of *L. monocytogenes*. Reducing the hydrolysis temperature of P-Cru by 14 °C resulted in a 2 percentage unit decrease in DH and some differences in the peptide composition. Nevertheless, the antimicrobial activity (in situ) was not significantly impacted, decreasing the viable count of *L. monocytogenes* by ~1-log and retarding their growth for 21 days at 4 °C. Although the color of the product was visibly altered, leading to more saturated reddish and yellowish tones and reduced brightness, the discoloration of the hydrolysates can be addressed. This biopreservation approach holds promise for other meat products and contributes to the circular economy concept of the meat industry by valorizing slaughterhouse blood and producing new antilisterial compounds.

## 1. Introduction

The rising demand for fresh, convenient, and additive-free food products has led to an increase in the sales of RTE food around the world such as deli meats [1]. Although RTE meat products are pathogen-free after a proper thermal treatment during processing, they can easily be recontaminated in post-processing stages (e.g., segmentation, slicing, repackaging, and others) by pathogens from equipment and the environment, particularly *L. monocytogenes* [2,3]. This Gram-positive bacterium is one of the most problematic foodborne pathogens since it can cause listeriosis. This illness is considered as a relatively rare infection, but is of great concern to public health due to its clinical severity, resulting in high hospitalization rates (>90%) and mortalities (20–30%) in large outbreaks, especially for immunocompromised people [4]. Indeed, around 273 stillbirths globally annually are attributed to listeriosis [5]. Moreover, *L. monocytogenes* is also a serious concern for RTE meat products since it can grow in a wide range of temperatures (0–45 °C) and pH values (4.3–9.6), as well as thrive in the presence or absence of oxygen, low moisture content, and high salt concentrations [6,7,8]. Furthermore, its ability to form biofilms fosters the persistence of this pathogen on equipment and in the environment, and hence, constitutes a higher risk of post-processing cross-contamination [9,10,11]. Consequently, both raw meat and RTE meat products have been considered important vehicles for *L. monocytogenes* transmission [11]. For instance, pork and pork products such as pork charcuterie have been implicated in listeria outbreaks worldwide [12]. In addition, many studies reported the prevalence of this pathogen in slaughterhouses and processing plants, as well as in pork meat and processed pork products such as ham [13,14,15,16,17,18]. Therefore, controlling this pathogen in both manufacturing plants and food is a major challenge for the meat industry.

Among the different strategies for the control of food spoilage microorganisms and pathogens in meat products, the use of biopreservatives of natural origin, such as antimicrobial peptides, has gained increasing interest from industrialists and researchers [19,20]. In this context, an approach has emerged for the preservation of meat products using antimicrobial peptides derived from meat industry by-products, particularly slaughterhouse blood. Over thirty antimicrobial peptides have been found in peptic hydrolysates of bovine hemoglobin (Hb-B), the main protein of red blood cells (~90%), which have shown inhibitory activity against spoilage microorganisms and/or pathogens [21,22,23,24,25,26,27]. Some of these peptides are homologous or present similar physicochemical characteristics to other hemoglobin (Hb) sources such as porcine due to their amino acid sequence similarity (86.5% identity for α-subunit and 82.3% identity for β-subunit) [28,29]. In addition, it has been suggested that this approach fits the circular economy concept since it is possible to recycle a part of the proteins of slaughterhouse blood to preserve meat and meat products [30]. For instance, Przybylski et al. [21] reported that neokyotorphin (TSKYR), a small peptide (653 Da) with antimicrobial and antioxidant activities derived from the bovine hemoglobin α chain and homologous in porcine hemoglobin (Hb-P), can be used as a preservative to extend the shelf-life of minced beef. These authors also observed that this peptide reduced the growth of total coliforms in beef as well as its lipid oxidation by up to 60%. In the same way, this approach can contribute to solving the serious management problem that represents this by-product for the meat industry such as the large volume produced, its low added value, its potential environmental contamination, and the additional disposal costs [31]. However, the use of hemoglobin hydrolysates for the control of pathogens in meat products is an unexplored field to date. To the best of our knowledge, no in situ studies have been reported in meat products for the control of *L. monocytogenes* or other pathogens by using hemoglobin hydrolysates. Nevertheless, recent studies have also attributed antilisterial activity to peptide hydrolysates of porcine hemoglobin and porcine cruor [28,29]. As demonstrated in our previous study [28], the peptic hydrolysates of Hb-P showed antilisterial activity against *Listeria ivanovii*, whereas the antilisterial activity of peptide hydrolysates from P-Cru was demonstrated by Zouari et al. [29] against *Listeria innocua*.

Therefore, this study aimed to evaluate the potential of the peptic hydrolysates of porcine hemoglobin as a biopreservative to control *L. monocytogenes* in pork cooked ham. The main objectives were (1) to characterize the physicochemical properties of the peptic hydrolysates of Hb-P and P-Cru and their peptide population, (2) to compare their in vitro and in situ (challenge test in pork cooked ham) antibacterial activities against *L. monocytogenes*, and (3) to evaluate the impact of the hydrolysates on the color of pork cooked ham.

## 2. Materials and Methods

### 2.1. Materials

Porcine hemoglobin (Hb-P, purity > 98%) was acquired from Sigma-Aldrich Chemical Corporation (St. Louis, MO, USA) and derived from the same batch of porcine blood. Porcine cruor was produced from fresh porcine blood collected from the Olymel slaughterhouse (Olymel S.E.C./L.P., Vallée-Jonction, QC, Canada). Ethylenediaminetetraacetic acid tetrasodium salt dihydrate (EDTA, Sigma-Aldrich, Oakville, ON, Canada) was used as an anticoagulant. Pepsin from porcine gastric mucosa (EC 3.4.23.1, 3200–4500 units/mg protein) was also purchased from Sigma-Aldrich Chemical. HCl and NaCl used in the production of the hydrolysates were acquired from Fisher Scientific (Nepean, ON, Canada). Sodium tetraborate from Fisher Scientific (Ottawa, ON, Canada), sodium dodecyl sulfate (SDS) from Bio-Rad Laboratories Inc. (Tokyo, Japan), *o*-phthalaldehyde (OPA) from Sigma-Aldrich (St. Louis, MO, USA), DL-leucine from Alfa Aesar (Shanghai, China), and β-mercaptoethanol from Sigma-Aldrich (St. Louis, MO, USA) were utilized to determine the degree of hydrolysis. The reagents employed in the RP-UPLC-MS/MS analyses were of analytical grade and procured from Sigma-Aldrich. For the antimicrobial tests, tryptic soy broth (TSB) from BD Bacto™ (Franklin Lakes, NJ, USA), Hardy Diagnostics CRITERION™ Agar (Santa Maria, CA, USA), peptone from BD Bacto™ (Sparks, MD, USA), 1.07004 Oxford *Listeria* Selective Agar (MilliporeSigma Canada Ltd., Oakville, ON, Canada), 1.07006 Oxford *Listeria* selective supplement (MilliporeSigma Canada Ltd., Oakville, ON, Canada), and pediocin from Laboratoire Innodal Inc. (Longueuil, QC, Canada) were employed.

### 2.2. Production of Hb-P and Porcine Cruor Hydrolysates

#### 2.2.1. Hb-P Hydrolysate

The Hb-P hydrolysate was prepared following the method described by Sanchez-Reinoso et al. [28] with minor adjustments. Briefly, a 1% (*w*/*w*) Hb-P solution was prepared in Milli-Q water by stirring overnight (150 rpm) at 10 °C. Subsequently, the pH was adjusted at 3.0 using HCl and transferred to a shaking water bath (VWR International, LLC, Radnor, PA, USA) set at a temperature of 37 °C and subjected to continuous agitation at 80 rpm. Pepsin was then added (enzyme/substrate (E/S) ratio of 1:11 mol/mol) and incubated for 3 h by keeping constant the temperature, agitation, and pH. Finally, enzymatic hydrolysis was stopped by increasing the pH to 10.0 with NaOH. The final pH of the hydrolysate was adjusted to 7 to avoid a significant alteration of the pH of the target product (ham, pH = 6.2), which was determined using the Health Canada [32] method MFHPB-03. Three independent samples were prepared. Finally, the samples were lyophilized and stored at −20 °C until further analyses.

#### 2.2.2. Porcine Cruor Hydrolysate

Fresh porcine blood was obtained from different healthy pigs on a single collection day, which was quickly treated with EDTA (1 g/L) as the animal bled to prevent clotting. Then, cruor was recovered by the centrifugation of porcine blood on Beckman Coulter Avanti JE (Avanti J-E, Beckman Coulter, Brea, CA, USA) at 8000 rpm for 15 min. Five sequential centrifugation steps were applied: an initial centrifugation step to obtain the solid fraction (cruor), followed by four subsequent rinsing steps with NaCl at 0.9% (*w*/*v*). The cruor was finally freeze-dried, vacuum-packed, and stored at −20 °C for further enzymatic hydrolysis.

The P-Cru hydrolysis was performed from an initial 2% (*w*/*w*) cruor protein solution prepared with Milli-Q water and stirred overnight at 10 °C. The peptic hydrolysis of P-Cru was also carried out as was described for the Hb-P hydrolysate with some modifications. The pH of the solution was adjusted to 3.0 using HCl. The solution was tempered at 23 °C and maintained at this temperature during the hydrolysis process with constant stirring (80 rpm). Then, pepsin was added at an E/S ratio of 1/15 mol/mol, and the pH was kept constant at 3 with HCl. After 3 h of enzymatic hydrolysis, the pepsin was inactivated by increasing the pH to 10.0 with NaOH. The pH of the final hydrolysate was also adjusted to 7.0. Finally, the hydrolysate was freeze-dried and stored at −20 °C until further analyses. Higher initial protein concentration (2% *w*/*w*), lower hydrolysis temperature (room temperature ~23 °C), and lower E/S ratio (1:15) were employed for the hydrolysis of P-Cru to reduce water, energy consumption, and enzyme consumption, as well as to be closer to real industrial conditions for a potential scale-up application. The hydrolysis was conducted in triplicate.

### 2.3. Determination of the Degree of Hydrolysis

The DH of the Hb-P and P-Cru hydrolysates was determined by an *o*-phthalaldehyde (OPA) spectrophotometric assay reported by Church et al. [33] with some modifications. The OPA reagent was prepared as described by Sanchez-Reinoso et al. [34]. A volume of 150 µL of each sample and 3.0 mL of the OPA solution were mixed by inversion in a 4.5 mL acrylic cuvette. Then, the absorption was measured at 340 nm after 2 min of incubation at room temperature in an Agilent 8453 UV–visible spectroscopy system (Hewlett–Packard Company, Waldbronn, Germany). The DH was determined according to Equation (1). A standard curve (R^2^~0.999) with DL-leucine concentrations ranging from 0.75 to 3.00 mM was used to calculate the number of free amino groups. The values were determined from three repetitions.

(1) $$DH=\frac{\left(h-h_{0}\right)}{h_{tot}}\times 100$$

where DH is the degree of hydrolysis (%), h the number of free amino groups released by cleavage of peptide bonds during the enzymatic hydrolysis of hemoglobin (mEq/g protein), h_0_ the number of free amino groups of pure Hb-P or P-Cru before pepsin addition (mEq/g protein), and h_tot_ the total number of peptide bonds for hemoglobin (8.3 mEq/g protein [35]).

### 2.4. Characterization of Hydrolysates by Using RP-UPLC-MS/MS and Bioinformatic Tools

#### 2.4.1. Peptide Profiles of Hydrolysates

The peptide population of the Hb-P and P-Cru hydrolysates was compared by RP-UPLC-MS/MS, following the methodology described by Abou-Diab et al. [30] with minor adjustments. A 1290 Infinity II UPLC (Agilent Technologies, Santa Clara, CA, USA) was used, consisting of a multi-sampler (G7167B), a binary pump (G7120A), an in-line degasser, and a variable wavelength detector (VWD G7114B) set to 214 nm. Prior to analysis, the Hb-P and P-Cru hydrolysates were filtered on 0.22 µm polyvinylidene fluoride (PVDF) filters and injected onto a Poroshell 120 EC-C18 column (2.1 × 100 mm i.d., 2.7 µm, Agilent, Santa Clara, CA, USA). The column was operated at a flow rate of 500 µL/min and maintained at 23 °C. A linear gradient employing solvent A (LC-MS grade water with 0.1% formic acid) and solvent B (LC-MS grade acetonitrile with 0.1% formic acid) was applied, with solvent B transitioning from 1% to 65% over 25 min, then ramping up to 95% and holding for 5 min before returning to initial conditions.

For peptide identification, an LC-MS/MS analysis was conducted using an Agilent 6560 ion mobility quadrupole time-of-flight (IM-Q-TOF) mass spectrometer (Agilent, Santa Clara, CA, USA). The mass spectrometer operated in the positive mode, with a scan range of 100–3200 *m*/*z*, and the signals were acquired at an extended dynamic range of 2 GHz. Nitrogen gas was used as both the drying gas (13.0 L/min and 150 °C) and the nebulizer gas (30 psig). The capillary, nozzle, and fragmentor voltages were set to 3500, 300, and 400 V, respectively. Instrument calibration was performed using an ESI-L low-concentration tuning mix (G1969-85000, Agilent Technologies, Santa Clara, CA, USA). Data acquisition and analysis were conducted using the Agilent Mass Hunter Software package (LC/MS Data Acquisition, Version B.08.00 and Qualitative Analysis for IM-MS, Version B.07.00 Service Pack 2 with BioConfirm Software). In addition, the unidentified peptides were assessed in the FindPept database (http://ca.expasy.org/tools/findpept.html (accessed on 9 July 2023)) to establish the possible peptide sequences comparing the molecular masses with the sequences of the alpha chain (α-globin) and beta chain (β-globin) of porcine hemoglobin.

Furthermore, peptide quantification based on the UV peak areas recovered from the RP-UPLC chromatograms at 214 nm was carried out according to Kosters et al. [36]. The molar concentration of the peptides was calculated using Equation (2).

(2) $$C_{pep}=1\times 10^{6}\left(\frac{A_{214}}{\varepsilon_{214}\;L\;V_{inj}\;K_{cell}}\right)f$$

where C_pep_ (µM) is the concentration of peptide, A_214_ (AU min) the RP-UPLC peak area of the absorbance at 214 nm, ε_214_ (1/M cm) the molar extinction coefficient at 214 nm of each peptide according to Kuipers and Gruppen [37] method, L (cm) the path length of the UV cell which was 1 cm, V_inj_ (μL) the injection volume, *f* (μL/min) the flow rate, and K_cell_ the cell constant. K_cell_ was established beforehand to be 0.06 through calibration using neokyotorphin (TSKYR) solutions at known concentrations under the same analysis conditions [28].

In certain RP-UPLC peaks, the presence of co-eluting peptides was observed, in which case a further correction of C_pep_ was applied by dividing the peak area by the number of peptides present in the same RP-UPLC peak. In this instance, it was presumed that the peptides contributed equally to absorbance [38].

#### 2.4.2. Identification of Potential Antimicrobial Peptides

Potential antimicrobial peptides were identified by in silico analysis evaluating their physicochemical and structural characteristics. The physicochemical properties of the identified peptides (molecular mass, GRAVY index, total hydrophobic residues, and net charge at pH 7.0) were determined using the network antimicrobial peptide database (http://aps.unmc.edu/AP/ (accessed on 9 July 2023)) [39], whereas their secondary structures were predicted using the network protein sequence analysis (https://prabi.ibcp.fr/ (accessed on 9 July 2023)) [40] Additionally, the sequences identified in the Hb-P and P-Cru hydrolysates were also compared with the sequences previously reported in the literature with proven antibacterial activity against *Listeria* ssp. by basic local alignment search tool (BLAST) [41].

### 2.5. Protein Content of Hydrolysates

The freeze-dried Hb-P and P-Cru hydrolysates were rehydrated at known concentrations based on protein content for in vitro antilisterial activity characterization and in situ test. For this purpose, Duma’s combustion method was used to determine the total nitrogen content of the lyophilized Hb-P and P-Cru hydrolysates using a rapid Micro N cube (Elementar, Langenselbold, Germany). The protein content was estimated using a nitrogen-to-protein conversion factor of 6.25 [42].

### 2.6. Evaluation of In Vitro Antilisterial Activity of Hydrolysates

The antimicrobial activity of the hydrolysates was tested against three different serotypes of *L. monocytogenes*: ATCC 19112 (serotype 2(1/2c)), ATCC 19115 (serotype 4b), and ATCC 15313 (serotype 1/2a). They were selected due to their importance in human health issues, as they have been involved in the outbreaks of listeriosis [7].

#### 2.6.1. Agar Well Diffusion Method

An agar well diffusion assay was performed in order to evaluate the antilisterial activity of the Hb-P and P-Cru hydrolysates according to Sanchez-Reinoso et al. [28]. Briefly, 25 mL of soy tryptic agar (0.75% *w*/*w* agar) previously melted and tempered at 45 °C was separately inoculated with 250 µL of an 18 h subculture of each target serotype (~1 × 10^6^ colony-forming unit (CFU)/mL). The inoculated culture medium was deposited in Petri dishes, solidified at room temperature, and punctured with a sterile pipette to prepare the wells. Then, the wells were loaded with 80 µL of each hydrolysate (40 mg/mL). Sterile water was used as a negative control, whereas pediocin (150 µg/mL) was employed as a positive control. The plates were incubated at 37 °C for 24 h and the diameter of the clean zone of growth inhibition was measured. The mean ± standard error of the diameter of growth inhibition (mm) was reported, which was calculated from three repetitions.

#### 2.6.2. Determination of Minimum Inhibitory Concentration (MIC) and Minimum Bactericidal Concentration (MBC)

The MIC of the Hb-P and P-Cru hydrolysates for the three selected serotypes of *L. monocytogenes* was determined by the liquid growth inhibition assay. The assay was performed in a 96-well polystyrene microplate (Becton Dickinson Labware, Sparks, MD, USA) [28]. The microplates were full of a twofold serial dilution of each hydrolysate (20–0.04 mg/mL) in tryptic soy broth. Then, the wells were inoculated with 50 μL of the target strains with a concentration between 0.5 and 1.0 × 10^6^ CFU/mL. The microplates were incubated at 37 °C for 24 h and the absorbance at 595 nm after incubation was measured by using an Infinite F200 PRO photometer (Tecan US Inc., Durham, NC, USA). The MIC was identified as the minimum concentration of the hydrolysate that effectively halted the growth of the target strains, resulting in an optical density equivalent to that of the uninoculated tryptic soy broth (negative control). In addition, MBC was also determined by spreading 10 µL of the solution from the wells that exhibited no growth after incubation onto tryptic soy agar (TSA) Petri dishes, followed by incubation at 37 °C for 24 h. The MBC was defined as the lowest dilution without growth, which was also employed to establish the bactericidal (MFC/MIC ≤ 4) or bacteriostatic (MFC/MIC > 4) effect of the hydrolysates [34].

### 2.7. L. monocytogenes In Situ Challenge Test on Refrigerated Pork Cooked Ham

#### 2.7.1. Preparation of *L. monocytogenes* Inoculum

A three-time sub-culture (1% *v*/*v*) of *L. monocytogenes* (frozen glycerol stock at −80 °C) from the three serotypes in tryptic soy broth (TSB, Difco Laboratories, Sparks, MD, USA) at 37 °C with 24 h intervals was employed for the in situ test. Cells were collected by centrifugation at 7500× *g* for 15 min at 4 °C (Heraeus Multifuge 1S-R centrifuge; Thermo Scientific, Waltham, MA, USA), washed twice with sterilized phosphate-buffer saline (PBS) (0.01 M phosphate, pH 7.2), and re-suspended in 10 mL of PBS to obtain a final concentration of approximately 10^6^ CFU/mL. Finally, the *L. monocytogenes* cocktail was prepared by combining equal volumes of the three different serotype strains selected [43].

#### 2.7.2. Inoculation and Storage of Pork Cooked Ham

The treatment and inoculation of pork cooked ham were carried out according to Tahiri et al. [43] with some modifications. Thin slices of pork cooked ham (~19.9 ± 0.1 g) were purchased from a local commercial supermarket (Quebec City, QC, Canada) and treated immediately. One surface of each pork cooked ham slice was treated with 1 mL of the Hb-P hydrolysate solution (50 mg/mL), P-Cru hydrolysate solution (50 mg/mL), and pediocin solution as a positive control (125 µg/mL) by using sterile L-shaped cell spreaders (Fisher Scientific, Pittsburg, PA, USA). These values represent 10 times the MIC necessary to inhibit *L. monocytogenes* ATCC 19112 in tryptic soy broth (the highest MIC observed for the three target serotypes). Additionally, inoculated and non-inoculated pork cooked ham controls were included. In the case of the inoculated control, 1 mL of sterile distilled water was used instead of a biopreservative solution. The ham slices were kept in a laminar-flow biological safety cabinet for ~10 min to dry off excess liquid. Then, 1 mL of the *L. monocytogenes* cocktail (10^6^ CFU/mL) was spread on each one to give a final inoculum of 10^4^ CFU/g of pork cooked ham. Finally, the samples were packed individually in Whirl-Pak sterile bags and stored at 4 °C for 21 days. The samples were collected on day 0 (immediately after applying the treatments) and periodically with intervals of 3 days for microbiological analyses (enumeration of *L. monocytogenes*). Three repetitions were carried out for each treatment, including 3 pork cooked ham slices per treatment per storage period.

#### 2.7.3. Enumeration of *L. monocytogenes*

The enumeration of *L. monocytogenes* in the pork cooked ham slices was carried out according to the Health Canada method MFLP-74 for the enumeration of *L. monocytogenes* in foods with slight modifications [44]. A 1:5 dilution of the sample in peptone water (0.1% *w*/*v*) was prepared by adding 80 mL of peptone water into the Whirl-Pak sterile bags containing the whole slices. Then, the samples were stomached for 2 min at 250 rpm by using a Stomacher 400 circulator (Seward, Therfford, Norfolk, UK). The decimal serial dilutions of the homogenate were prepared in 0.1% (*w*/*v*) peptone water. Then, 100 μL of the dilutions were spread onto the surfaces of Petri dishes with selective media 1.07004 Oxford *Listeria* Selective Agar with 1.07006 Oxford *Listeria* selective supplement and the plates were incubated at 37 °C for 48 h. The *L. monocytogenes* colonies (green colonies with a surrounded opaque halo) were counted, and the results were expressed as Log CFU/g. The analyses were performed in triplicate and the values were reported as means ± standard errors with a detection limit < 50 CFU/g.

### 2.8. Color Parameters

The color of the treated and untreated pork cooked ham slices was measured with a Minolta colorimeter CR-300 (Konica Minolta Inc., Osaka, Japan) with 8 mm aperture, C illuminant, 0 observers, and set to the Commission International de I’Eclairage (CIE) L*a*b* color space system. This system represents color in a three-dimensional L*a*b* space: coordinate L* represents lightness on a scale from 0 (black) to 100 (white), coordinate a* represents red (positive values, +a*) and green (negative values, −a*), and coordinate b* represents yellow (positive values, +b*) and blue (negative values, −b*). The CIE L*a*b* color coordinates were measured in triplicate with five readings for each sample. Moreover, the parameters of chroma (C*), hue (h*), and total color difference (ΔE*, color difference with respect to the samples without biopreservative) were calculated using Equations (3)–(5), respectively [45].

(3) C*=a*2+b*212

(4) h*=tan−1b*a*

(5) $$\Delta E=\sqrt{{\left({\Delta L}^{*}\right)}^{2}+{\left({\Delta a}^{*}\right)}^{2}+{\left({\Delta b}^{*}\right)}^{2}}$$

### 2.9. Statistical Analysis

The Hb-P and P-Cru hydrolysates were prepared according to a completely random arrangement. For this purpose, the day of slaughter was considered as a fixed effect (a single collection day of blood), whereas the slaughtered animals were considered as a random term (randomly selected pigs). The day of hydrolysis was also considered as a random term, which was carried out on three different days. No effect of hydrolysis day was present since all the Hb-P and P-Cru samples remained lyophilized and stored at −20 °C until the day of hydrolysis, and the same conditions of rehydration (agitation, temperature, and time) before peptic hydrolysis were used. All the hydrolysates were compared in terms of DH, peptide population, and in vitro antilisterial activity through Student’s t-test at 95% confidence. A completely randomized design was also used in the in situ challenge test with four treatments (the Hb-P hydrolysate, P-Cru hydrolysate, pediocin solution, and untreated sample as control), and determinations were conducted at days 0, 3, 6, 9, 12, 15, 18, and 21 of storage. In this case, the treatment and the storage day were considered fixed effects since all the samples were inoculated, collected, and analyzed at the same points in time. No significant interaction was observed between the treatment and the storage day. The statistical analysis of the in situ challenge test was performed using one-way ANOVA at 95% confidence followed by Tukey’s multiple range tests. The results were reported as mean ± standard error and the software Statistix v 9.0 (Analytical Software, Tallahassee, FL, USA) was employed for the statistical analysis. Heat maps of the peptide population were plotted by using RStudio Version 1.4, while the other figures were plotted by using the SigmaPlot integrated software Version 12.0 (Systat Software, Inc., San Jose, CA, USA).

## 3. Results and Discussion

### 3.1. Degree of Hydrolysis of Hydrolysates

**Figure 1:**
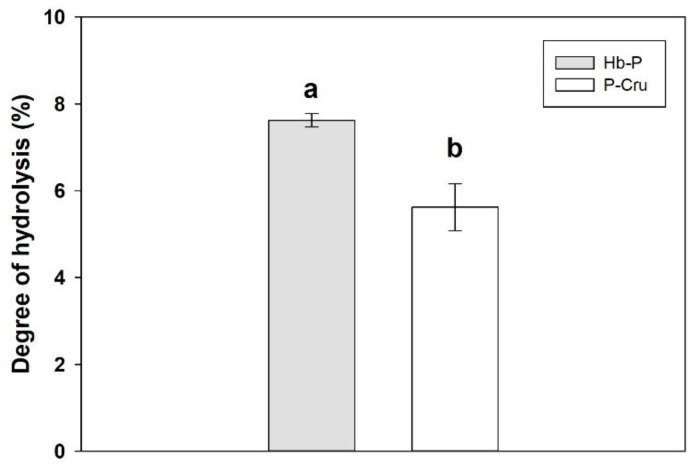
Comparison of the DH of the porcine hemoglobin (Hb-P) and porcine cruor (P-Cru) hydrolysates after 3 h of peptic hydrolysis. The error bars represent the standard error. The means with the same lowercase letter indicate that both means are not significantly different by Student’s *t*-test at the 5% probability level.

Figure 1 depicts the DH of the Hb-P and P-Cru hydrolysates after 3 h of peptic hydrolysis. As can be seen, the Hb-P hydrolysate had a significantly higher DH than that obtained by the P-Cru hydrolysate (*p*-value < 0.05), achieving values of 7.6 ± 0.2 and 5.6 ± 0.5%, respectively. These results are expected due to the difference in the temperature and E/S ratio employed during the enzymatic hydrolysis. Pepsin has an optimal activity at 37 °C, which is adversely affected by its variation [46]. In the case of P-Cru, the peptic hydrolysis was performed at 23 °C to reduce energy consumption and to be closer to industrial conditions for potential industrial-scale production. This difference of 14 °C lower for the P-Cru hydrolysate compared to the temperature used for the Hb-P hydrolysate (37 °C) had a negative impact on the pepsin activity, which was evidenced by a decrease in the final DH by 2 percentage units. In addition, the increase in the E/S ratio for P-Cru (1:15 mol/mol) may also be responsible for the lower DH due to the higher availability of the substrate to be hydrolyzed. The reduction in temperature and enzyme amount required for the enzymatic hydrolysis could represent a significant energy and cost saving in an industrial application. Nonetheless, these parameters affect the enzymatic efficiency as can be observed. The difference in DH could also suggest a difference in the peptide population of the hydrolysates, since a higher DH might represent a higher number of peptides released, probably of lower molecular masses. It is important to note that a higher DH does not necessarily represent higher antimicrobial activity as this could negatively impact the hydrolysis of intermediate antimicrobial peptides by hydrolyzing them into smaller peptides of no antimicrobial importance [28]. This further reinforces the importance of conducting a UPLC-MS/MS analysis, which includes the assessment of peptide concentration, in order to investigate the influence of temperature on the peptide composition of hemoglobin hydrolysates.

### 3.2. Characterization of Hydrolysates by Using RP-UPLC-MS/MS and Bioinformatic Tools

#### 3.2.1. Peptide Profiles of Hydrolysates

**Figure 2:**
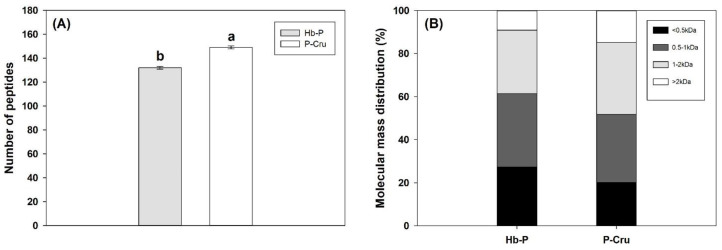
Number of peptides (**A**) and molecular mass distribution (**B**) identified by the RP-UPLC−MS/MS of the peptic hydrolysates produced from porcine hemoglobin (Hb-P) and porcine cruor (P-Cru). The error bars represent the standard error. The means with the same lowercase letter indicate that both means are not significantly different by Student’s *t*-test at the 5% probability level.

The comparison of the number of peptides and the molecular mass distribution of the Hb-P and P-Cru hydrolysates are shown in Figure 2A and 2B, respectively. Significant differences between the Hb-P and P-Cru hydrolysates were observed in both the number of peptides and molecular mass distribution (*p*-value < 0.05). P-Cru presented 17 additional peptides compared to the Hb-P hydrolysate. This result may appear to be contrary to that observed for the DH. Nonetheless, this can be explained by the principle involved in the method to measure the DH (OPA assay), which considers both the peptides and free amino acids of the hydrolysates [28]. Conversely, certain dipeptides and free amino acids were excluded from the analysis of the peptide population due to the limitations in the sensitivity of the MS/MS method. This assumption can be supported by the results observed in the molecular mass distribution of the peptides identified by RP-UPLC−MS/MS. Another explanation may be the behavior of peptide concentration, since a higher concentration of specific peptides may reflect a higher DH regardless of the number of peptides [34]. As can be noticed in Figure 2B, the Hb-P hydrolysates presented a higher presence of minor peptides (<1 kDa) as well as a lower presence of large peptides (>1 kDa) compared to the P-Cru hydrolysate. The Hb-P hydrolysate had 7 percentage units more peptides < 0.5 kDa and 3 percentage units more peptides 0.5–1 kDa than the P-Cru hydrolysate, as well as 4 and 6 percentage units fewer peptides of 1–2 kDa and >2 kDa, respectively. This may suggest a greater enzymatic action of pepsin on pure Hb-P than P-Cru, probably due to the hydrolysis temperature.

**Figure 3:**
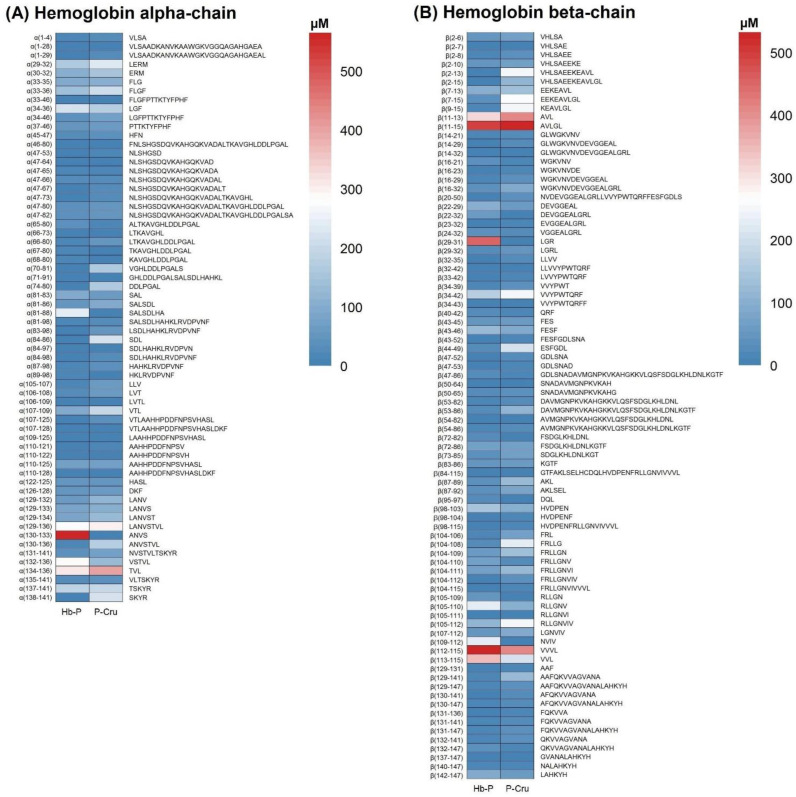
Heat map based on the peptide concentration (µM) of the identified peptides in the porcine hemoglobin (Hb-P) and porcine cruor (P-Cru) hydrolysates. The identified compounds are labeled by their sequence and location in the sequences of the alpha chain (**A**) or beta chain (**B**). The treatments and peptides are visualized in each column and row, respectively. The values in the diagram were calculated from the average of the three replicate samples.

The heat maps of the peptide concentration of the Hb-P and P-Cru hydrolysates at 3 h of peptic hydrolysis are presented in Figure 3. In general, the hydrolysates presented high similarity in the presence of peptides. A total of 116 peptides with identical sequences were identified in common. However, the concentration of multiple peptides differed among the hydrolysates. A total of 23 peptides identified in the P-Cru hydrolysates were absent in the Hb-P hydrolysates, including 11 peptides from the hemoglobin α-subunit (α(1–28), α(33–46), α(46–80), α(47–67), α(70–81), α(71–91), α(74–80), α(84–86), α(84–97), α(107–128), and α(138–141)) and 12 peptides from the hemoglobin β-subunit (β(2–13), β(2–15), β(14–29), β(14–32), β(20–50), β(32–42), β(43–52), β(50–64), β(54–82), β(84–115), β(98–115), and β(104–115)). The concentration of most of these peptides was in the range of 1.5–33.1 µM, except for the peptides α(70–81), α(74–80), α(138–141), β(2–13), and β(2–15) that presented a concentration higher than 115 µM. This may explain the behavior of a higher number of peptides in the P-Cru hydrolysate despite their lower DH since most of them did not present a high concentration (<33.1 µM). In addition, most of these peptides, except α(138–141) and β(2–13), were also absent or very low in concentration (α(1–28), α(71–91), α(71–91), and β(50–64)) in a 6.5% DH peptic hydrolysate of Hb-P produced under similar conditions (pH 3, 37 °C for 3 h) [34]. This supports the hypothesis that these peptides are intermediates and their concentration could decrease over a longer hydrolysis time (>3 h), which is also associated with a lower DH for the P-Cru hydrolysate. In contrast, a total of nine peptides identified in the Hb-P hydrolysates were absent in the P-Cru hydrolysates, in which three peptides were derived from the hemoglobin α-subunit (α(81–88), α(106–109), and α(130–133)) and six peptides were derived from the hemoglobin β-subunit (β(22–32), β(29–31), β(72–82), β(95–97), β(105–109), and β(109–112)). The concentration of these peptides varied between 10.5 and 565.0 µM. This would be another explanation for the lower number of peptides for the Hb-P hydrolysate despite its higher DH since a lower number of peptides with high concentration could lead to a higher DH. As examples, it can be mentioned that the peptides α(81–88), β(29–31), and α(130–133) presented concentrations of 246.1, 452.3, and 565.0 µM, respectively.

These results are consistent with the behavior of DH, peptide number, and molecular mass distribution, supporting the hypothesis that the reduction in the optimal temperature of pepsin during hydrolysis generates some variations in the peptide profile and peptide concentration of the Hb-P and P-Cru hydrolysates.

#### 3.2.2. Identification of Potential Antimicrobial Peptides

Complementary to the peptide population analysis, an in silico analysis of the structural properties of the peptides was performed in order to identify potential antimicrobial peptides. For this purpose, structural features such as the percentage of hydrophobic residues, net charge at pH 7, GRAVY index, and secondary structure prediction were considered. Peptides with a high proportion of hydrophobic residues, a net positive charge from +1 to +6, as well as a helix or random coil structure were considered potentially antimicrobial since they can be structured in contact with the biological membrane of microorganisms, developing inactivation mechanisms by cell membrane disruption or enzymatic activity at the intracellular level [28]. These physicochemical and structural characteristics are typical of cationic antimicrobial peptides. A total of 44 potential antimicrobial peptides were identified: 10 derived from the α-subunit and 34 derived from the β-subunit (Table S1). Similarities were observed in the presence of potential antimicrobial peptides reported by Sanchez-Reinoso et al. [28], as well as the presence of peptides with possible antilisterial activity. In the case of the hemoglobin alpha chain, the peptides α(1–28), α(1–29), α(33–46), α(34–46), α(37–46), and α(137–141) derived from Hb-P or P-Cru are analogous (>80% sequence identity) to the peptides α(1–28), α(1–29), α(33–46), α(34–46), α(37–46), and α(137–141) derived from bovine hemoglobin (Hb-B). The antimicrobial activity of the latter peptides against *L. innocua* was demonstrated by Nedjar-Arroume et al. [47]. In addition, the same authors also demonstrated the antilisterial activity of the β(140–145) peptide (LAHRYH) found in Hb-B, as well as peptides including LAHRYH as terminal sequence such as β(114–145), β(121–145), and β(126–145). This first peptide (LAHRYH) has an 83% sequence similarity to peptide β(142–147) peptide (LAHKYH), which could indicate that both this peptide and the other peptides with the same terminus could also exhibit antilisterial activity such as β(129–147), β(130–147), β(131–147), β(132–147), and β(140–147).

Additionally, some anionic peptides with potential antilisterial activity were also identified. For this purpose, a comparison of the sequence of the anionic peptides found in the Hb-P and P-Cru hydrolysates versus the anionic antimicrobial peptides derived from Hb-B previously reported in the literature [25,47,48,49] with antibacterial activity against *L. innocua* was carried out (Table S2). Among them, 12 anionic peptides were identified in the Hb-P and/or P-Cru hydrolysates with high sequence similarity compared to the peptides derived from Hb-B.

**Figure 4:**
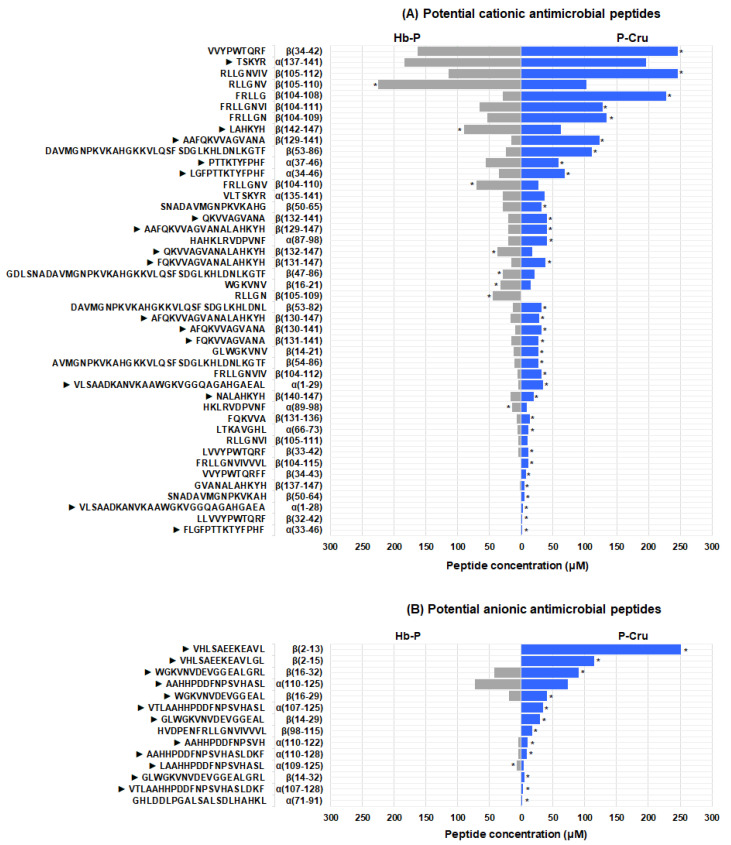
Tornado plot of the concentration (µM) of the potential cationic (**A**) and anionic (**B**) antimicrobial peptides identified in the Hb-P (pure porcine hemoglobin, gray bars) and P-Cru (porcine cruor, blue bars) hydrolysates by RP-UPLC-MS/MS. The bars with asterisks (*) indicate a significantly higher peptide concentration according to Student’s *t*-test at the 5% probability level. ► indicates the peptides with similar sequences to the previously reported Hb-B peptides with proven antimicrobial activity against *L. innocua*.

A graphical comparison (tornado plot) of the concentration of the potential cationic and anionic antimicrobial peptides identified in the peptic hydrolysates of Hb-P and P-Cru was also performed (Figure 4). As can be seen in Figure 4, certain significant differences in both the profile of potential antimicrobial peptides and their concentration can be appreciated. Regarding the potential cationic antimicrobial peptides (Figure 4A), some differences were present in the peptides with low concentration (<11 µM), such as the absence of the peptides α(1–28), β(50–64), β(32–42), α(33–46), and β(104–115) in the Hb-P hydrolysate or the absence of the peptide β(105–109) in the P-Cru hydrolysate. In addition, a group of peptides could also be observed whose concentration was at least 0.5-fold higher for the Hb-P hydrolysate (β(142–147), β(105–110), β(16–21), β(132–147), and β(104–110)) or 0.5-fold higher for the P-Cru hydrolysate (α(87–98), β(130–141), β(104–109), β(131–147), β(129–141), β(104–108), α(1–29), β(105–112), β(53–82), β(53–86), β(34–42), β(129–147), β(104–111), α(34–46), and β(104–112)). However, the Hb-P and P-Cru hydrolysates also presented peptides in common with similar contents such as α(137–141), β(140–147), α(135–141), β(132–141), β(131–136), β(50–65), β(131–141), α(89–98), α(37–46), β(47–86), and β(33–42). As mentioned above, these differences can be explained by the difference in the hydrolysis temperature, which could also affect the antimicrobial activity of the final hydrolysates. In order to elucidate the impact of this variable on the antimicrobial activity against *L. monocytogenes*, further in vitro and in situ antimicrobial tests were performed.

Regarding the potential anionic antimicrobial peptides, the P-Cru hydrolysate presented a significantly higher concentration in 11 of the peptides identified compared to the Hb-P hydrolysate (Figure 4B). This would suggest that these peptides could be intermediates and would be hydrolyzed into smaller peptides after 3 h of peptic hydrolysis at optimal temperature (37 °C). Moreover, a higher presence of these peptides could favor the antibacterial activity of the final hydrolysate. Therefore, it would be important to consider the hydrolysis time and hence the DH to obtain the highest antibacterial activity against *L. monocytogenes*.

### 3.3. In Vitro Antilisterial Activity of Hydrolysates

The antimicrobial activity against three different serotypes of *L. monocytogenes* was evaluated prior to the challenge test to establish the concentration necessary for the treatment of the cooked pork ham slices, which was defined as 10-fold higher than the MIC. The results of the diameter of growth inhibition, MIC, and MBC are presented in Table 1. Also, the zones of growth inhibition can be observed in Figure S1. As can be seen, both the Hb-P hydrolysate and P-Cru hydrolysate presented inhibition for the three target *L. monocytogenes* serotypes ranging from 12.0 to 13.7 mm. Furthermore, no significant differences in the diameters of growth inhibition were observed between the Hb-P and P-Cru hydrolysates nor between the different serotypes evaluated (*p*-value > 0.05). Conversely, differences in MIC were observed for both substrate type (Hb-P or P-Cru) and serotypes, which ranged from 0.6 to 5.0 mg/mL. The Hb-P and P-Cru hydrolysates presented the same MIC value for serotype 2 (1/2c) (MIC = 5.0 mg/mL) and serotype 4b (MIC = 5.0 mg/mL), which were the most resistant and sensitive serotypes to inhibition, respectively. As for serotype 1/2a, the Hb-P hydrolysate (MIC = 1.25 mg/mL) was two times lower than that observed for the P-Cru hydrolysate (MIC = 2.5 mg/mL). This would suggest that the pure Hb-P peptic hydrolysate had a stronger antimicrobial activity against that serotype, which might be due to the difference in peptide population. As could be observed in the peptide profile of the hydrolysates, the Hb-P hydrolysate presented a higher concentration of some peptides such as β(142–147), β(105–109), β(105–110), β(16–21), β(132–147), and β(104–110), which were not reported previously as sequences with antilisterial activity according to the literature. This could suggest the stronger antimicrobial activity of these peptides against the latter serotype. Moreover, the difference in the MIC values between the serotypes could be due to the variations in the surface antigens of these serotypes. Serotypes 2 (1/2c) (ATCC 19112), 4b (ATCC 19115), and 1/2a (ATCC 15313) differ by a specific combination of somatic and flagellar surface antigens [50], which could influence the initial binding (interactions with cell membranes) and efficacy of antimicrobial peptides. According to the results, a concentration of 50 mg/mL was selected for the challenge test, corresponding to a 10-fold higher concentration of the highest MIC found for the three serotypes evaluated (5.0 mg/mL for serotype 2 (1/2c)), which was defined according to the literature [51].

### 3.4. L. monocytogenes Challenge Test of Refrigerated Pork Cooked Ham

**Figure 5:**
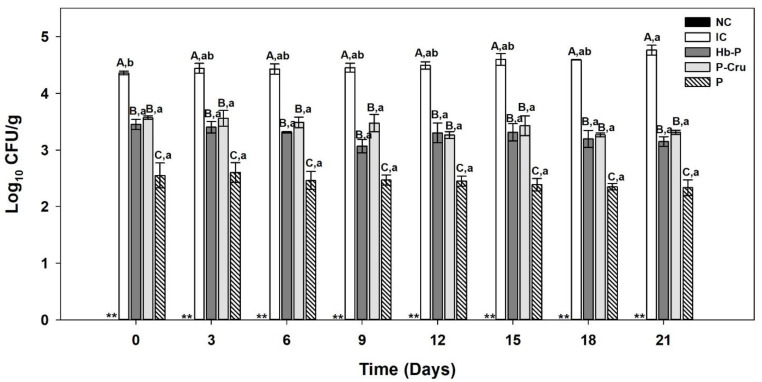
Evolution of the viable count of *L. monocytogenes* added to the pork cooked ham stored at 4 °C for 21 days: non-inoculated (NC), inoculated (IC), porcine hemoglobin (Hb-P) hydrolysate, porcine cruor (P-Cru) hydrolysates, and pediocin (P). The error bars indicate the standard errors of the repeated treatments (n = 3). The means ± standard errors with different uppercase letters within the same group of bars are significantly different according to Tukey’s test at the 5% probability level. The means ± standard errors for the same treatment with lowercase letters are significantly different over time according to Tukey’s test at the 5% probability level. The values for NC (**) were below the detection limit (<50 CFU/g).

The evolution of *L. monocytogenes* viable count during storage at 4 °C of the pork cooked ham is shown in Figure 5. *L. monocytogenes* was not detected in the control samples (NC), indicating that the samples were free of *L. monocytogenes* contamination and the count was that introduced by the in situ inoculation. Moreover, the different treatments displayed different profiles of *L. monocytogenes* inhibition. In the absence of a biopreservative, *L. monocytogenes* grew during storage and reached 4.8 log CFU/g after 21 days, increasing by 0.4 log CFU/g compared to the initial day (4.4 log CFU/g). Regarding the treated samples, *L. monocytogenes* viable counts showed an initial reduction after the application of biopreservatives (Hb-P hydrolysate, P-Cru hydrolysates, or pediocin), remaining significantly lower than the untreated sample (negative control) during the storage time (*p*-value < 0.05). The samples treated with the Hb-P and P-Cru hydrolysates presented a similar behavior in the evolution of the viable count of *L. monocytogenes*. The application of the Hb-P and P-Cru hydrolysates resulted in an immediate 0.9-log and 0.8-log reduction, respectively. This difference increased slightly throughout the storage, reaching values of 1.0–1.2 log reduction after 21 days. Likewise, an initial reduction of 1.8 log CFU/g was observed for pediocin, which also increased slightly to 2.0 log CFU/g by the last day of storage. These results suggest that the application of porcine hemoglobin hydrolysates at 50 mg/mL reduces the viable number of *L. monocytogenes* by approximately 1-log in cooked pork ham refrigerated at 4 °C, as well as generating a bacteriostatic effect during 21 days of storage at the same temperature.

In the regulatory framework for ready-to-eat (RTE) foods, tolerance limits have been established for *L. monocytogenes* that vary between countries from the zero-tolerance policy (0 CFU in 25 g) for all RTE foods to tolerance < 100 CFU/g for RTE foods that do not support growth [52]. Canada follows the latter approach, as stated in the policy on *L. monocytogenes* in RTE foods [53]. At first sight, the challenge test results would suggest that the Hb-P and P-Cru hydrolysates would not accomplish this requirement. However, it is important to mention that the initial concentration of inoculum used in the challenge test exceeded the levels observed in the reported meat product outbreaks. Hence, considering the requirements for the use of microbial agents for the control of *L. monocytogenes* indicated in this policy, the Hb-P and P-Cru hydrolysates could be acceptable since no more than 2-log increase throughout the stated shelf-life of the product was observed. In addition, the separation, purification, and/or isolation of the peptides of interest (e.g., membrane and electro-membrane processes) could also be explored as alternatives to enhance the target antilisterial activity as demonstrated by Przybylski et al. [54] for neokyotorphin derived from bovine hemoglobin. The authors achieved a 75-fold increase in the purity of this peptide compared to the initial hydrolysate by electrodialysis with ultrafiltration membrane (EDUF), which has been shown to inhibit the growth of spoilage microorganisms and delay meat rancidity [21]. However, further studies are required to validate this effect on the antilisterial activity of Hb-P and P-Cru hydrolysates.

### 3.5. Impact of Hydrolysates on the Color Properties of Ham

**Figure 6:**
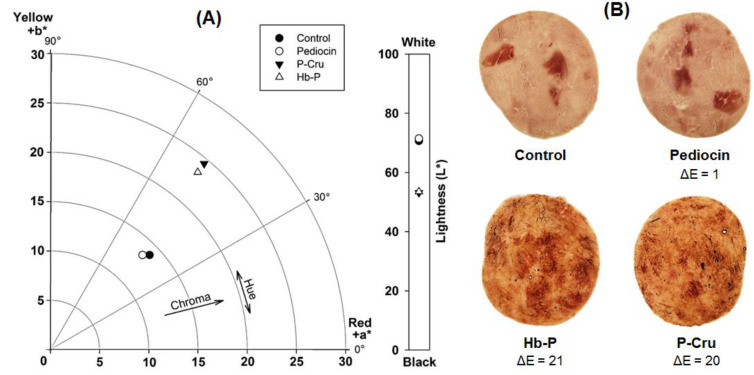
CIE L*a*b* color coordinates (**A**) and color appearance (**B**) of the treated and untreated pork cooked ham slices with biopreservatives. Total color difference (ΔE) scale: 0–0.5 = are not perceptible, 0.5–1.5 = are slightly perceptible, 1.5–3.0 = are noticeable, 3.0–6.0 = are well visible, >6.0 = are very visible [45]. Hb-P is the porcine hemoglobin hydrolysate and P-Cru is the porcine cruor hydrolysate.

Color is one of the most important sensory attributes in consumer purchasing decisions. Therefore, the characteristic color of the food product should not be altered for the application of hemoglobin hydrolysates as biopreservatives. The appearance of the pork cooked ham slices treated with biopreservatives (Hb-P hydrolysate, P-Cru hydrolysate, or pediocin) and untreated, as well as the color coordinates (CIE L*a*b*), are presented in Figure 6. As can be observed and as expected, the change in coloration is evident for the samples treated with the Hb-P and P-Cru hydrolysates compared to the control (untreated sample). The untreated pork ham presented brightness values of 70.6 ± 1.8 as well as positive values of a* (10.1 ± 1.2) and b* (9.6 ± 0.4), indicating a tendency towards yellowish and reddish coloration (Figure 6A). Similar values of the L*a*b* coordinates were observed for the samples treated with pediocin. In the same way, the samples treated with the Hb-P and P-Cru hydrolysates also showed positive values of the a* and b* coordinates. However, treatment with the hydrolysates decreased the lightness and increased chroma, indicating darker-colored samples with more saturated red and yellow colors. The color change in the treated samples is due to the presence of heme groups in the raw hydrolysates. Heme is a red macrocyclic compound containing a central atom of divalent ferrous iron (Fe^2+^) [55]. The heme groups remain after the enzymatic hydrolysis of Hb-P and P-Cru, increasing the pigment concentration on the surface of the ham treated with hydrolysates, and consequently increasing color saturation and reducing light refraction. These differences were also evident in the total color difference (Δ*E*), which indicated that the samples treated with the Hb-P and P-Cru hydrolysates presented a very visible difference in relation to the untreated sample (Figure 6B). In contrast, the samples treated with pediocin showed slightly visible differences.

Color could represent a limitation regarding the applicability of hemoglobin hydrolysates since the typical coloration produced by the heme groups forming hemoglobin significantly impacts the typical color of the product. Nevertheless, two approaches can be considered to solve this concern. In the first place, hemoglobin hydrolysates could be used in reddish-colored meat products in which their color attributes are not significantly affected. In this approach, both the bioprotective effect against *L. monocytogenes* and the additional supply of iron provided by the heme groups could also be considered. A second approach could consider an additional stage of the discoloration of the hydrolysates by the chemical acid precipitation of the heme groups. The discoloration of hemoglobin hydrolysates has been previously studied in bovine and porcine hemoglobin hydrolysates [48,56]. Nonetheless, it has been observed that the discoloration of hemoglobin hydrolysates may negatively impact the antilisterial activity. Abou-Diab et al. [56] observed that the discolored hydrolysates from bovine hemoglobin presented a higher MIC (2.5 mg/mL) compared to the raw hydrolysates (1.25 mg/mL), increasing by two times the MIC against *L. monocytogenes* (ATCC 19112) after discoloration. It could be explained by the fact that some peptides with antilisiterial activity can be precipitated with heme groups during discoloration. Indeed, a study recently reported by Cournoyer et al. [48] established that the discoloration of the peptic hydrolysates of P-Cru reduced by up to 12-fold the antifungal activity due to the precipitation of some specific peptides during this process. Thus, further studies are required to verify the impact of discoloration on the antimicrobial activity of the peptic hydrolysates of Hb-P and P-Cru against *L. monocytogenes*.

## 4. Conclusions

The use of the peptic hydrolysates of Hb-P as a method of the biocontrol of *L. monocytogenes* in pork cooked ham was evaluated in situ. To the best of our knowledge, this work presents the first approach that adopts peptic hydrolysates from hemoglobin for the control of this pathogen in ready-to-eat meat products. It was demonstrated that it is possible to obtain peptides with antilisterial activity against serotypes involved in outbreaks directly from P-Cru, among which new, previously unreported sequences were identified. Furthermore, it is possible to use a lower hydrolysis temperature without significantly affecting the bacteriostatic action for 21 days of storage at 4 °C. This benefits the feasibility of this approach by requiring less energy consumption and provides a basis for industrial scale-up. It was also established that hemoglobin hydrolysates modified product color visually appreciably. Thus, it is suggested as a perspective to study the impact of discoloration on the antimicrobial activity against *L. monocytogenes*., as well as studies at higher storage temperatures to estimate the impact of temperature fluctuation during ham commercialization on antilisterial activity. Additionally, it would be important to explore other sources of hemoglobin from different animal species and their applications in fresh meat and other meat products. Similarly, the exploration of the impact of raw and discolored hemoglobin hydrolysates on antimicrobial activity against spoilage microorganisms, lipid oxidation, and the sensory properties of the products seems to be relevant for future studies.

## Figures and Tables

**Table 1:** Determination of MIC, MBC, MBC/MIC of peptic hydrolysates of Hb-P and P-Cru.

Strain	Serotype	Hb-P Hydrolysate				P-Cru Hydrolysate			
		Inhibition Halo (mm)	MIC (mg/mL)	MBC (mg/mL)	MBC/MIC	Inhibition Halo (mm)	MIC (mg/mL)	MBC (mg/mL)	MBC/MIC
ATCC 19112	2 (1/2c)	12.0 ± 0.0 ^A,b^	5.00 ± 0.00 ^A,a^	5.00 ± 0.00 ^A,b^	1.00 ± 0.00	12.0 ± 0.0 ^A,b^	5.00 ± 0.00 ^A,a^	5.00 ± 0.00 ^A,b^	1.00 ± 0.00
ATCC 19115	4b	13.7 ± 0.3 ^A,a^	0.63 ± 0.00 ^A,c^	2.50± 0.00 ^B,c^	4.00 ± 0.00	13.3 ± 0.3 ^A,a^	0.63 ± 0.00 ^A,c^	5.00 ± 0.00 ^A,b^	8.00 ± 0.00
ATCC 15313	1/2a	12.7 ± 0.3 ^A,ab^	1.25 ± 0.00 ^B,b^	>20.00 ± 0.00 ^A,a^	>16.00 ± 0.00	12.3 ± 0.3 ^A,ab^	2.50 ± 0.00 ^A,b^	>20.00 ± 0.00 ^A,a^	>8.00 ± 0.00

Means and standard errors were calculated from three repetitions. The means in columns followed by the same uppercase letter are not significantly different between the serotypes according to Tukey’s test at the 5% probability level. The means in rows for the same variable followed by the same lowercase letter are not significantly different between the hydrolysates according to Student’s *t*-test at the 5% probability level.

## Data Availability

The original contributions presented in the study are included in the article, further inquiries can be directed to the corresponding author.

## Supplementary Materials

The following supporting information can be downloaded at: https://www.mdpi.com/article/10.3390/foods13152394/s1, Table S1: Potential cationic antimicrobial peptides from Hb-P and P-Cru identified by RP-HPLC-MS/MS after 3 h of peptic hydrolysis. Table S2: Comparison of potential anionic antimicrobial peptides identified in hydrolysates of Hb-P/P-Cru and Hb-B by basic local alignment search tool (BLAST). Figure S1: Growth inhibition zones of porcine hemoglobin (Hb-P) and porcine cruor (P-Cru) peptic hydrolysates against *L. monocytogenes* strains ATCC 19112 (A), ATCC 19115 (B), and ATCC 15313 (C). C− is the negative control (sterilized distilled water) and C+ is the positive control (pediocin). Hb-P1, Hb-P2, and Hb-P3 are Hb-P hydrolysate repetitions 1, 2, and 3, respectively. P-Cru1, P-Cru2, and P-Cru3 are P-Cru hydrolysate repetitions 1, 2, and 3, respectively.
