# Supplementary material for: Biocontrol Strategy of Listeria monocytogenes in Ready-to-Eat Pork Cooked Ham Using Peptic Hydrolysates of Porcine Hemoglobin
Source: Foods. 2024 Jul 29;13(15):2394. doi: 10.3390/foods13152394 (PMC11311599; doi:10.3390/foods13152394)
Supplement: Supplementary file 1 [file foods-13-02394-s001.zip › foods-3081769-supplementary.pdf]

Supplementary Material S1

Table S1. Potential cationic antimicrobial peptides from Hb-P and P-Cru identified by RP-HPLC-MS/MS after 3 h of peptic hydrolysis.

Location	Sequence	MM (Da)	ε (M^{-1} cm^{-1})	GRAVY index[a]	Total hydrophobic residues (%)[a]	Net charge at pH 7[a]	Structure (%)[b]
α(1-28)	VLSAADKANVKAAWGKVGGQAGAHGAEA	2633.4	60234	0.000	50	+1.25	53.57% RC
α(1-29)	VLSAADKANVKAAWGKVGGQAGAHGAEAL	2746.5	61202	0.131	52	+1.25	62.07% RC
α(33-46)	FLGFPTTKTYFPHF	1701.9	48879	0.064	36	+1.25	78.57% RC
α(34-46)	LGFPTTKTYFPHF	1554.8	42756	-0.146	31	+1.25	84.62% RC
α(37-46)	PTTKTYFPHF	1237.6	34751	-0.810	20	+1.25	90.00% RC
α(66-73)	LTKAVGHL	837.5	11854	0.675	50	+1.25	50.00% RC
α(87-98)	HAHKLRVDPVNF	1431.8	28778	-0.550	42	+1.5	75.00% RC
α(89-98)	HKLRVDPVNF	1223.7	21775	-0.520	40	+1.25	100.00% RC
α(135-141)	VLTSKYR	865.5	11219	-0.457	29	+2	100.00% RC
α(137-141)	TSKYR	653.3	9285	-2.240	0	+2	100.00% RC
β(14-21)	GLWGKVNV	871.5	35861	0.388	50	+1	37.50% RC
β(16-21)	WGKVNV	701.4	33949	-0.050	50	+1	100.00% RC
β(32-42)	LLVVYPWTQRF	1420.8	51991	0.573	55	+1	54.55% RC
β(33-42)	LVVYPWTQRF	1307.7	51023	0.250	50	+1	60.00% RC
β(34-42)	VVYPWTQRF	1194.6	50055	-0.144	44	+1	100.00% RC
β(34-43)	VVYPWTQRFF	1341.7	56178	0.150	50	+1	100.00% RC
β(47-86)	GDLSNADAVMGNPKVKAHGKKVLQSFSDGLKHLDNLKGTF	4236.2	62028	-0.500	35	+2.5	75.00% AH
β(50-64)	SNADAVMGNPKVKAH	1537.8	22351	-0.573	40	+1.25	80.00% RC
β(50-65)	SNADAVMGNPKVKAHG	1594.8	23295	-0.563	38	+1.25	62.50% RC
β(53-82)	DAVMGNPKVKAHGKKVLQSFSDGLKHLDNL	3245.7	47169	-0.507	37	+2.5	66.67% AH
β(53-86)	DAVMGNPKVKAHGKKVLQSFSDGLKHLDNLKGTF	3679.0	56164	-0.512	35	+3.5	67.65% AH
β(54-86)	AVMGNPKVKAHGKKVLQSFSDGLKHLDNLKGTF	3563.9	55183	-0.421	36	+4.5	63.64% AH
β(104-108)	FRLLG	604.4	9105	1.100	60	+1	100.00% RC
β(104-109)	FRLLGN	718.4	10164	0.333	50	+1	100.00% RC
β(104-110)	FRLLGNV	817.5	11130	0.886	57	+1	71.43% RC
β(104-111)	FRLLGNVI	930.6	12098	1.338	63	+1	48.39% RC
β(104-112)	FRLLGNVIV	1029.6	13064	1.656	67	+1	77.78% RC
β(104-115)	FRLLGNVIVVVL	1340.9	15964	2.258	75	+1	41.67% RC
β(105-109)	RLLGN	571.3	4041	-0.160	40	+1	100.00% RC
β(105-110)	RLLGNV	670.4	5007	0.567	50	+1	100.00% RC
β(105-111)	RLLGNVI	783.5	5975	1.129	57	+1	100.00% RC
β(105-112)	RLLGNVIV	882.6	6941	1.513	63	+1	100.00% RC
β(129-141)	AAFQKVVAGVANA	1244.7	16905	1.008	69	+1	69.23% AH

β(129-147)	AAFQKVVAGVANALAHKYH	1994.1	38186	0.374	58	+2.5	84.21% AH
β(130-141)	AFQKVVAGVANA	1173.7	15950	0.942	67	+1	33.33% RC
β(130-147)	AFQKVVAGVANALAHKYH	1923.0	37231	0.294	56	+2.5	83.33% AH
β(131-136)	FQKVVA	690.4	10116	0.933	67	+1	33.33% RC
β(131-141)	FQKVVAGVANA	1102.6	14995	0.864	64	+1	27.27% RC
β(131-147)	FQKVVAGVANALAHKYH	1852.0	36276	0.206	53	+2.5	82.35% AH
β(132-141)	QKVVAGVANA	955.5	8872	0.670	60	+1	40.00% RC
β(132-147)	QKVVAGVANALAHKYH	1704.9	30153	0.044	50	+2.5	75.00% AH
β(137-147)	GVANALAHKYH	1179.6	25237	-0.191	45	+1.5	45.45% RC
β(140-147)	NALAHKYH	952.5	22372	-0.963	38	+1.5	62.50% RC
β(142-147)	LAHKYH	767.4	20358	-1.000	33	+1.5	100.00% RC

MM: Molecular mass. GRAVY index: grand average of hydropathicity index, which indicates the solubility of the peptides: positive GRAVY (hydrophobic) and negative GRAVY (hydrophilic). ε: molar extinction coefficient. RC: random coil. AH: Alpha helix.

[a] Values calculated by using the network antimicrobial peptide database (http://aps.unmc.edu/AP/) [1].

[b] Values predicted by using the network protein sequence analysis (https://prabi.ibcp.fr/) with secondary structure consensus prediction (MLRC, DSC, and PHD) [2].

References

1. Wang, G.; Li, X.; Wang, Z. APD3: the antimicrobial peptide database as a tool for research and education. *Nucleic Acids Research* **2015**, *44*, D1087-D1093, doi:10.1093/nar/gkv1278.
2. Combet, C.; Blanchet, C.; Geourjon, C.; Deléage, G. NPS@: network protein sequence analysis. *Trends in biochemical sciences* **2000**, *25*, 147-150, doi:10.1016/s0968-0004(99)01540-6.

Supplementary Material S2

Table S2. Comparison of potential anionic antimicrobial peptides identified in hydrolysates of Hb-P/P-Cru and Hb-B by basic local alignment search tool (BLAST) [1].

Hb-P/P-Cru hydrolysate			Hb-B hydrolysate			Query Length (AA)	Query Cover (%)	Identity (%)	Reference
**Location**	**Sequence**	**Net charge at pH 7**	**Location**	**Sequence**	**Net charge at pH 7**				
α(71-91)	GHLDDLPGALSALSDLHAHKL	-1.25	α(71-91)	GHLDDLPGALSALSDLHAHKL	-1,25	21	100	100	Cournoyer *et al.* [2]
α(110-128)	AAHHPDDFNPSVHASLDKF	-1.25				19	100	74	
α(110-125)	AAHHPDDFNPSVHASL	-1.25	α(110-128)	ASHLPSDFTPAVHASLDKF	-0.5	16	100	69	Adje *et al.* [3]
α(110-122)	AAHHPDDFNPSVH	-1.25				13	100	62	
α(107-128)	VTLAAHHPDDFNPSVHASLDKF	-1.25				22	100	77	
α(107-125)	VTLAAHHPDDFNPSVHASL	-1.25	α(107-128)	VTLASHLPSDFTPAVHASLDKF	-0.5	19	100	100	Adje *et al.* [4]
α(109-125)	LAAHHPDDFNPSVHASL	-1.25				17	100	100	
β(2-13)	VHLSAEEKEAVL	-1.75	β(1-13)	MLTAEEKAAVTAF	-1	12	75	78	
β(2-15)	VHLSAEEKEAVLGL	-1.75				14	64	78	
β(14-29)	GLWGKVNVDEVGGEAL	-2				16	87	93	Nedjar-Arroume *et al.* [5]
β(14-32)	GLWGKVNVDEVGGEALGRL	-1	β(1-30)	MLTAEEKAAVTAFWGKVKVDEVGGEALGRL	-1	19	89	94	
β(16-29)	WGKVNVDEVGGEAL	-2				14	100	93	
β(16-32)	WGKVNVDEVGGEALGRL	-1				17	100	94	
β(98-115)	HVDPENFRLLGNVIVVVL	-0.75	β(98-115)	HVDPENFRLLGNVIVVVL	-0.75	19	100	100	Cournoyer et al. [2]

AA: amino acids. Query length indicates the number of amino acids queried. Query cover indicates the percent of the query length that is included in the aligned segments.

References

1. NCBI Resource Coordinators. Database resources of the National Center for Biotechnology Information. *Nucleic Acids Research* **2013**, *42*, D7-D17, doi:10.1093/nar/gkt1146.
2. Cournoyer, A.; Thibodeau, J.; Ben Said, L.; Sanchez-Reinoso, Z.; Mikhaylin, S.; Fliss, I.; Bazinet, L. How Discoloration of Porcine Cruor Hydrolysate Allowed the Identification of New Antifungal Peptides. *Foods* **2022**, *11*, 4035.
3. Adje, E.Y.; Balti, R.; Lecouturier, D.; Kouach, M.; Dhulster, P.; Guillochon, D.; Nedjar-Arroume, N. Controlled Enzymatic Hydrolysis: A New Strategy for the Discovery of Antimicrobial Peptides. *Probiotics and Antimicrobial Proteins* **2013**, *5*, 176-186, doi:10.1007/s12602-013-9138-y.
4. Adje, E.Y.; Balti, R.; Kouach, M.; Dhulster, P.; Guillochon, D.; Nedjar-Arroume, N. Obtaining antimicrobial peptides by controlled peptic hydrolysis of bovine hemoglobin. *International Journal of Biological Macromolecules* **2011**, *49*, 143-153, doi:https://doi.org/10.1016/j.ijbiomac.2011.04.004.
5. Nedjar-Arroume, N.; Dubois-Delval, V.; Adje, E.Y.; Traisnel, J.; Krier, F.; Mary, P.; Kouach, M.; Briand, G.; Guillochon, D. Bovine hemoglobin: an attractive source of antibacterial peptides. *Peptides* **2008**, *29*, 969-977, doi:https://doi.org/10.1016/j.peptides.2008.01.011.

Supplementary Material S3

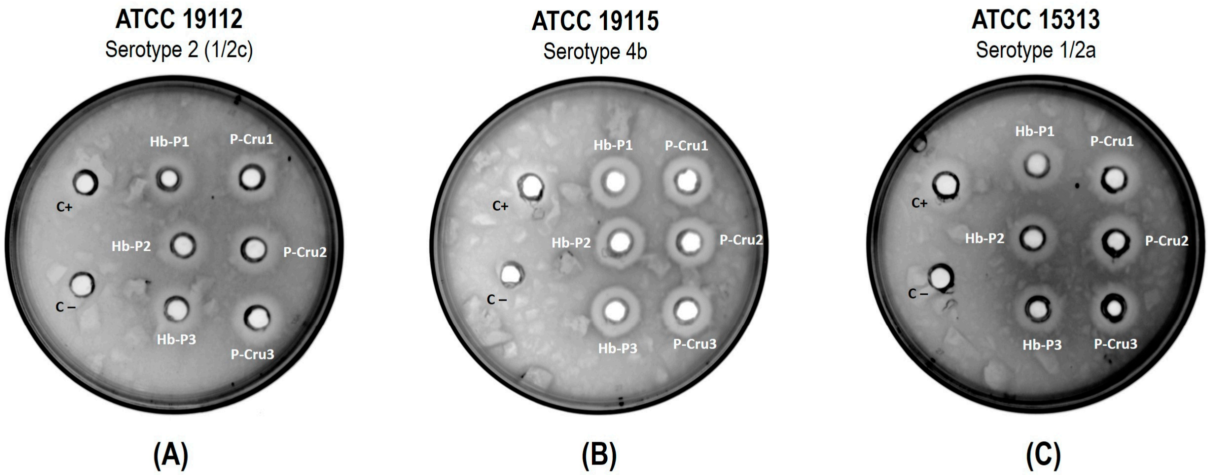

Figure S1. Growth inhibition zones of porcine hemoglobin (Hb-P) and porcine cruor (P-Cru) peptic hydrolysates against *L. monocytogenes* strains ATCC 19112 (A), ATCC 19115 (B), and ATCC 15313 (C). C – is the negative control (sterilized distilled water) and C+ is the positive control (pediocin). Hb-P1, Hb-P2, and Hb-P3 are Hb-P hydrolysate repetitions 1, 2, and 3, respectively. P-Cru1, P-Cru2, and P-Cru3 are P-Cru hydrolysate repetitions 1, 2, and 3, respectively.
